# Crashworthiness Study of Functional Gradient Lattice-Reinforced Thin-Walled Tubes under Impact Loading

**DOI:** 10.3390/ma17102264

**Published:** 2024-05-11

**Authors:** Zeliang Liu, Yuan Wang, Xi Liang, Wei Yu

**Affiliations:** 1 School of Civil Engineering and Mechanics, Yanshan University, Qinhuangdao 066004, China; wy36992021@163.com (Y.W.); ysulxi@163.com (X.L.); yuwei@ysu.edu.cn (W.Y.); 2 Hebei Key Laboratory of Mechanical Reliability for Heavy Equipments and Large Structures, Qinhuangdao 066004, China; 3 State Key Laboratory of Metastable Materials Science and Technology, Qinhuangdao 066004, China

**Keywords:** impact load, gradient lattice, thin-walled tubes, crashworthiness, energy absorption

## Abstract

Creating lightweight and impact-resistant box structures has been an enduring pursuit among researchers. A new energy-absorbing structure consisting of a bionic gradient lattice-enhanced thin-walled tube is presented in this article. The gradient lattice and thin-walled tube were prepared using selective laser melting (SLM) and wire-cutting techniques, respectively. To analyze the effects of gradient pattern, mass ratio, diameter range and impact speed on structural crashworthiness, low-speed impact at 4 m/s and finite element simulation experiments were conducted. The study demonstrates that the design of inward radial gradient lattice-reinforced thin-walled tubes can effectively enhance structure’s energy-absorption efficiency and provide a more stable mode of deformation. It also shows a 17.44% specific energy-absorption advantage over the uniformly lattice-reinforced thin-walled tubes, with no significant overall gain in peak crushing force. A complex scale evaluation method was used to determine the optimum structure and the structure type with the best crashworthiness was found to be a gradient lattice-filled tube with a thickness of 0.9 mm and a slope index of 10. The gradient lattice-reinforced thin-walled tube suggested in this investigation offers guidance for designing a more efficient thin-walled energy-absorption structure.

## 1. Introduction

Thin-walled tubes are extensively incorporated in energy-absorbing mechanisms across aerospace, transportation and marine sectors as they possess exceptional properties such as low weight, high energy-absorption efficiency and affordability [1,2]. To achieve energy absorption and dissipation under load, thin-walled tubes are subjected to a plastic deformation by progressive or symmetric pleating [3,4]. In recent years, there has been a significant amount of research conducted on materials used for thin-walled tubes, cross-sectional shapes and defect guidance, among other related topics [5,6,7,8]. The objective was to decrease the initial peak forces, enhance specific energy absorption and attain consistent and controlled deformation modes [9]. However, it is a challenge to achieve a high level of absorption energy simply by perforating the wall and changing the cross-sectional design.

Lightweight materials such as honeycomb sandwich, aluminum foam and lattice structures have been used to fill thin-walled structures to improve energy absorption [10,11]. However, the effectiveness of mixing tube travelling and energy absorption of the structure are limited due to issues with traditional fillers such as honeycomb and metal foam materials, which suffer from poor design ability and high randomness in preparation. As a novel lightweight structure, the point structure exhibits superior specific stiffness/strength, reduction of damping and vibration, cushioning, energy absorption and other functionalities [12]. The advancement of additive manufacturing technology has resulted in the creation of numerous lattice-reinforced filled structures possessing complex shapes [13]. For instance, BCC lattice-filled structures exhibit a 115% increase in energy absorption when compared to the combined absorption of the constituents in hybrid structures [10].

Despite the satisfactory energy-absorption properties of the aforementioned thin-walled tubes, researchers strive to enhance their capacity to absorb energy while maintaining a reduced mass [14,15,16,17]. The principle of bionics opens up novel research avenues for developing structures that are lighter in weight and possess higher energy-absorption capabilities. Drawing on the features that imitate the gradient distribution of the sargassum rod diameter, a novel functional gradient lattice infill tube has been created by researchers. This infill tube exhibits a specific energy-absorption rate that is 173.9% higher than that of a uniform lattice infill tube and displays variable stiffness along the entire length of the hybrid structure [12]. The gradient lattice was partitioned, resulting in a functional infill structure made from gradient lattice that was applied in ship structures. This resulted in a reduction of the maximum impact force by 60.01% [18].

In nature, radial gradient is the prevailing pattern in the cross-section of biological structures. However, there is a paucity of research on radial gradient lattice-enhanced thin-walled structures. Two types of radial gradient lattice-reinforced thin-walled tubes are designed in this study—inward radius decreasing mode (I-RLT) and outward radius decreasing mode (II-RLT). The impact resistance of these structures is investigated under impact loading conditions using experimental and finite element techniques. Moreover, the structure’s impact resistance was analyzed parametrically, considering the effects of gradient mode, wall thickness of the thin-walled tube, range of lattice radius and impact velocity.

## 2. Materials and Methods

### 2.1. Design of Components

**Figure 1:**
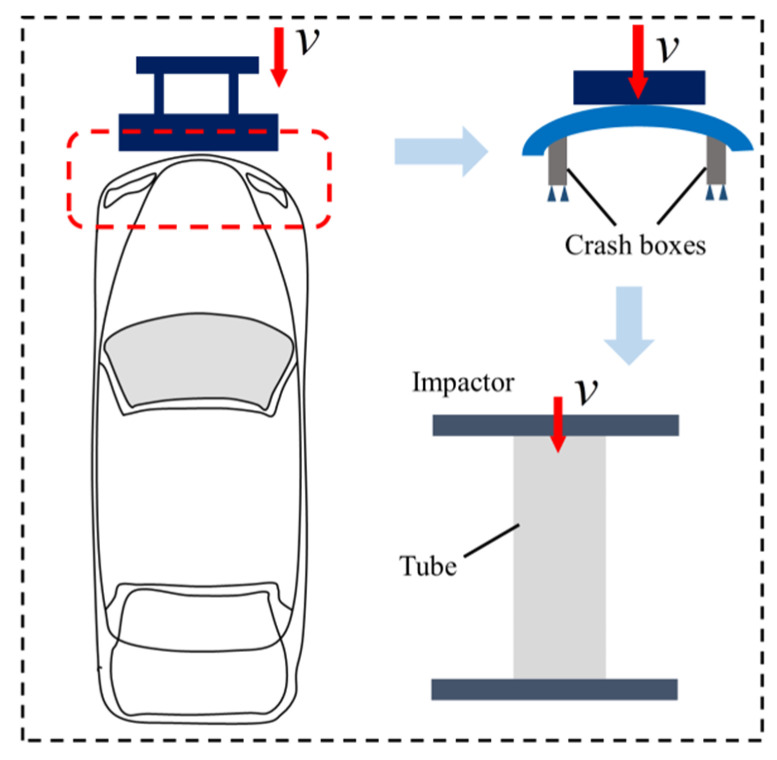
Schematic diagram and mathematical model of a car energy-absorption box.

After a frontal collision involving a car, the energy-absorbing structure is deformed plastically, leading to a reduction in damage to the vehicle and occupants. Figure 1 simplifies the impact to the energy-absorbing box, which is represented as a mathematical equation of a thin-walled tube under an axial impact load.

**Figure 2:**
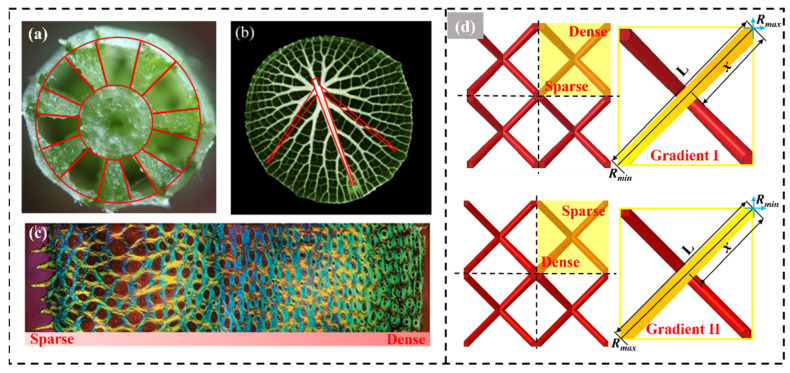
Organisms with gradient-distributed sparse–dense properties: (**a**) Horsetail grass and its cross-sectional diagram. Reproduced with permission [19] Copyright 2022, Elsevier. (**b**) Giant water lily leaf veins. Reproduced with permission [20] Copyright 2021, Elsevier. (**c**) Horseshoe hoof wall. Reproduced with permission [13] Copyright 2013, Elsevier. (**d**) New lattice structure.

Organisms are ‘naturally optimized’ to have unique composite structures that enable them to withstand a variety of external shocks. Among them, Figure 2a–c shows a typical gradient structure organism. Horsetail grass is relatively long and thin, but the complex internal configuration enables it to withstand wind loads and protect it from natural disasters. The form of gradient distribution of the leaf veins of the giant water lily facilitates the increase of the loading force of the leaves. The familiar ability to nail a horse’s hoof to an iron palm is attributed not only to the black cuticle on its surface, but also to the gradient-distributed tissues within the hoof that allow the horse to travel 10,000 miles a day, unaffected by rough and uneven mountain terrain. Figure 2d shows the front view of the bio-inspired lattice structures designed based on BCC lattices.

The gradient design of the lattice is achieved by deforming the eight rods in the unit cell towards the radius of the body center. The lattice density has a gradient distribution in the cross-section, following the law shown in Equation (1) [8,21,22,23,24,25].

(1) $$\rho (x)=\{\begin{array}{c} \rho_{max}-(\rho_{max}-\rho_{min}){\left(\frac{x}{L}\right)}^{m},\;\mathrm{gradient}\;\mathrm{pattern}\;\mathrm{of}\;I\\ \rho_{min}+(\rho_{max}-\rho_{min}){\left(\frac{x}{L}\right)}^{m},\;\mathrm{gradient}\;\mathrm{pattern}\;\mathrm{of}\;\mathrm{II} \end{array}$$

where ρ represents the density of the filled structure, with $\rho_{max}$ and $\rho_{min}$ being the maximum and minimum densities, respectively. The variable *L* refers to half of the diagonal length of the cross-section, while *x* denotes the length from the corner of the cross-section to its center at any position, as demonstrated in Figure 2d.

**Figure 3:**
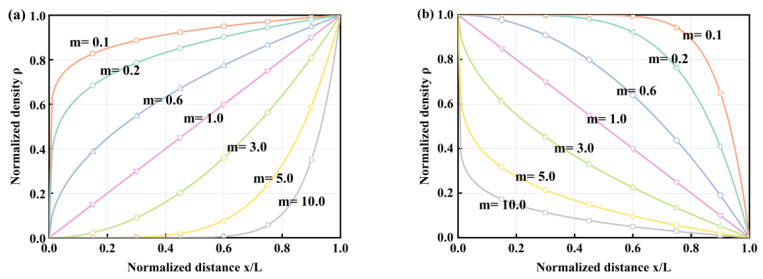
Variation of lattice density with normalized distance: (**a**) Gradient mode I; (**b**) Gradient mode II.

The exponent *m* of the gradient has an impact on both the lattice radius and the density distribution. Technical terms are defined upon their first usage. As can be seen in Figure 3a, when *m* goes from less than 1 to greater than 1, the function changes from a convex one to a concave one. Conversely, it transitions from being concave to convex in Figure 3b.

As the gradient exponent increases, the mass of the type I gradient pattern in the lattice increases gradually, while that of type II decreases. The initial structure consisted of a set of lattices, including 4 single cells, arranged perpendicularly to the cross-section, as shown in Figure 3b. When the lattice with two different gradient modes encounters the inner wall of the thin-walled tube, distinct interaction effects arise owing to the varying contact areas of the rods at the lattice’s end face and the different modes of action. The gradient structure design can create gradients at both the cellular and lattice levels. This allows for the formation of a single rod or an entire structure based on gradient principles.

In this paper, two asymptotic forms are designed in which the radius gradually increases and decreases from the center to the edge, and the gradient radius is controlled by expression 2.

(2) $$R(x)=\{\begin{array}{c} R_{max}-(R_{max}-R_{min}){\left(\frac{x}{L}\right)}^{m},\;\mathrm{gradient}\;\mathrm{pattern}\;\mathrm{of}\;I\\ R_{min}+(R_{max}-R_{min}){\left(\frac{x}{L}\right)}^{m},\;\mathrm{gradient}\;\mathrm{pattern}\;\mathrm{of}\;\mathrm{II} \end{array}$$

For individual cells, *R_max_* and *R_min_* represent maximum and minimum rod radius values, respectively. *M* is the gradient index, ranging from 0 to 10.

For the type I gradient pattern, the diameter of the rod is at its maximum at the edge of the lattice and at its minimum at the middle part of the lattice. This particular lattice is referred to as the inwardly decreasing radius lattice (I-RL). The type II gradient pattern is characterized by a lattice with a radius that decreases outwards (II-RL). It was placed in a thin-walled square tube measuring 60 mm in height and 30 mm × 30 mm in cross-section. The process of filling latticed arrays into a thin-walled tube to obtain a filled tube was recorded as I-RLT or II-RLT. In the latticed arrays of uniform structure (UX-RL), the letter X represents the diameter size. The data for all of the grating arrays are presented in Table 1.

### 2.2. Material Properties and Experimental Setups

**Figure 4:**
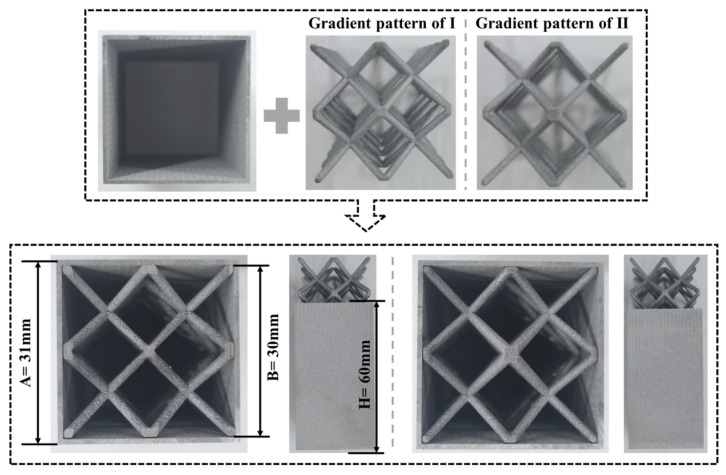
Experimentally prepared type I gradient and type II gradient lattice-filled tubes.

Experimentally prepared square thin-walled Al-1060 tubes and body-centered cubic AlSi10Mg lattices are shown in Figure 4. The internal dimensions are 30 mm × 30 mm, the thickness is 1 mm and the height of the tube is 60 mm. In this study, the figure displays square, thin-walled tubes made of Al-1060, produced using wire-cutting techniques. The new gradient lattices are made of an aluminum–silicon–magnesium alloy, AlSi10Mg and are body-centered cubic lattices produced using selective laser melting (SLM) techniques. The fabrication process is described below.

**Figure 5:**
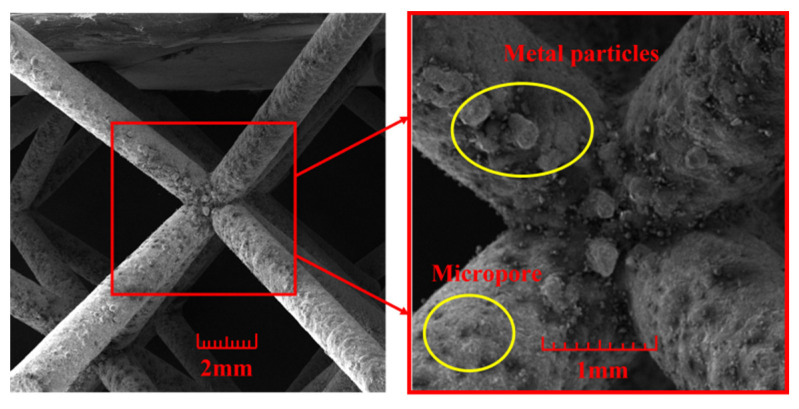
Microscopic surface of a new gradient lattice.

The lattice structure was produced using the iSLM600QN metal 3D printer from Zhongrui Technology (Suzhou, China). The fiber laser × 4 was used for laser type and the coating method was squeegee bi-directional powder laying. Further details can be found in Table 2. A microscopic view of the I-RL surface is shown in Figure 5. On the surface of the sample, irregularly distributed spherical micropores and powder particles are visible. This is likely due to gases not being precipitated in time or thermal diffusion occurring during the fabrication process. To eliminate residual stresses generated during the SLM process, the samples underwent heat treatment in air at 300 °C for 2 h. The manufacturing defect has impacted the experimental results, causing slight differences in the crashworthiness indices, such as specific energy absorption and initial peak load, when compared to the numerical simulations. To reduce this error, the specimens underwent heat treatment, resulting in experimental results that fell within acceptable limits.

**Figure 6:**
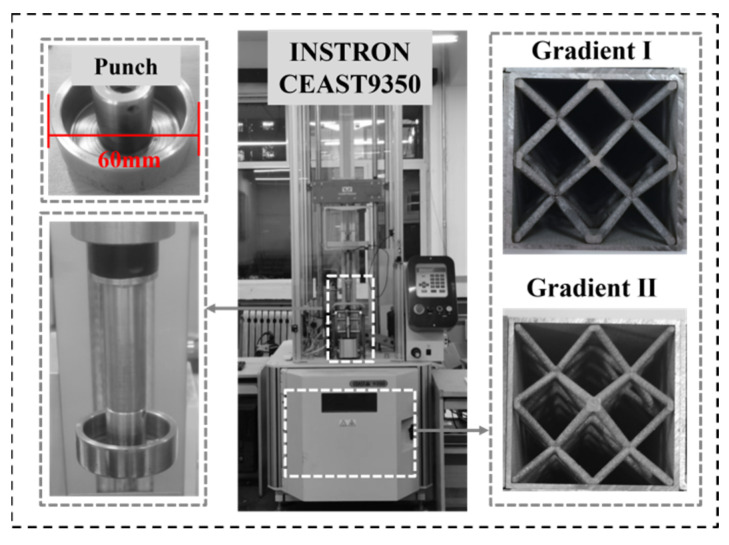
Dynamic impact experimental setup.

This paper presents the results of a dynamic impact (DI) test performed on a 60 mm filled tube to confirm the validity of the numerical simulation process. The INSTRON CEAST9350 Drop Hammer Impact Test System (Qinhuangdao, China) was used for the test, as shown in Figure 6. 

The instrument is versatile and can be used for a wide range of specimens, from composites to finished products. The collection gear and force transducer are attached to the impact hammer head. To adjust the system for different impact energies, simply adjust the counterweights and impact velocities. The head’s lower end is equipped with an automatic pneumatic rebound device that prevents secondary impact on the specimen caused by the head’s rebound. This feature makes it suitable for a wide range of impact tests, including tensile impact and breakdown. Before the test, the impact height is calibrated by adjusting the contact between the specimen and the punch using the adjustable height support in the closed box. During the dynamic impact, the total mass of the falling hammer is 21.2 kg, and the diameter of the flat punch position is 60 mm. The impact velocity is 4 m/s.

**Figure 7:**
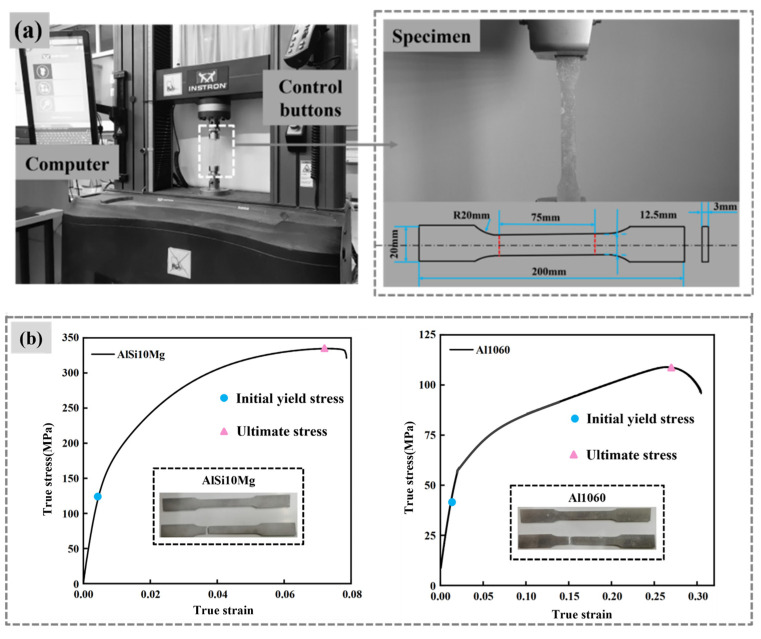
Test apparatus and mechanical properties of specimens: (**a**) Tensile test equipment; (**b**) True strain–stress curves.

The tube and lattice materials are chosen as Al-1060 and AlSi10Mg, respectively. The stress–strain curve and mechanical properties of the tube and lattice are given in Figure 7 and Table 3, respectively. The Zwick universal testing machine (Qinhuangdao, China) with a capacity of 100 kN, as shown in Figure 7a, was used for the tests. Tensile specimens were prepared strictly according to ASTM-E8M-04 and the tensile speed was deliberately set at 2 mm/min to ensure accurate and reliable results [26]. Both aluminum tubes and lattices were modelled using the elasto-plastic model, which is strain rate insensitive.

Table 3 shows the mechanical properties of Al-1060 and AlSi10Mg, and Figure 7b displays the true strain–stress curves [27,28]. For AlSi10Mg, the initial yield limit is determined by selecting the stress value corresponding to 1.5% plastic strain, in cases where there is no clear boundary between the elastic and yield phases. All members undergo heat treatment, which increases the yield limit and improves their mechanical properties, cutting and machining abilities.

### 2.3. Crashworthiness Index

The force–displacement graph provides a clear understanding of the energy-absorption characteristics of the structure. Other metrics used to assess this include total energy absorption (*EA*), Specific Energy Absorption (*SEA*), Mean Crush Force (*MCF*), Peak Crush Force (*PCF*), Crush Force Efficiency (*CFE*), etc., which are defined as follows:

(3) $$EA=\int_{0}^{\delta }F(\delta )d\delta$$

The structure’s impact resistance improves as the total absorbed energy increases. The equation above shows that *F*(*δ*) represents the instantaneous impact force, while *δ* represents the effective impact distance [29]. The effective distance can be calculated using the following equation:

(4) $$f=\frac{\int_{0}^{s}F(s)ds}{PCF}$$

(5) $$SEA=\frac{EA}{m}$$

(6) $$MCF=\frac{EA}{\delta }$$

(7) $$CFE=\frac{MCF}{PCF}$$

### 2.4. Finite Element Simulation and Experimental Verification

Finite element (FE) simulations are performed using nonlinear explicit FE code in Abaqus2019/Explicit. Across all analyses, the hybrid structures are situated between a fixed rigid plate and a moving rigid plate. It should be noted that the upper moving rigid plate was fixed in translation and rotation and only moved in the vertical direction and was subjected to displacement loads. The interactions among the rigid plates, tubes and lattice structures are defined by the general contact property. The contact behavior in the tangential direction is modelled using the penalty formulation. The friction coefficient for all contacts has been set to 0.25 [30]. Normal direction contact will be considered ‘hard contact’. The rigid plate in motion is connected to the structures through ‘tie’ constraints, which establish a master–slave node relationship. For both rigid plates, a reference point is defined. The reaction force history is recorded from the fixed rigid plate and the displacement history is recorded from the moving rigid plate. The 3D rigid bodies of the fixed and movable plates are modelled using four-node linear quadrilateral R3D4 elements. The lattice structures are modelled using four-node linear tetrahedral C3D4 elements, while the tubes are modelled with four-node S4R shell elements for reduced integration.

The square tubes are made of the aluminum alloy Al-1060 with mechanical properties of Young’s modulus *E* = 64.8 GPa, yield strength *σ_y_* = 118 MPa, mass density *ρ* = 2700 kg/m^3^ and Poisson’s ratio ν = 0.33. On the other hand, the type I gradient lattice structures are made of AlSi10Mg with mechanical properties of Young’s modulus *E* = 62.9 GPa, yield strength *σ_y_* = 325 MPa, mass density *ρ* = 2670 kg/m^3^ and Poisson’s ratio ν = 0.3.

**Figure 8:**
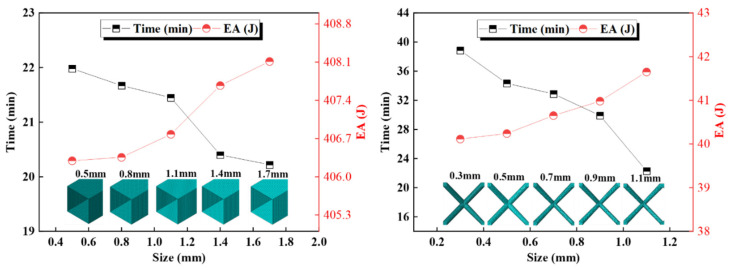
Mesh test for thin-walled tube and lattice.

The mesh test results showed that a global cell size of 0.5 mm was appropriate for the solid lattice structure and 0.8 mm for the thin-walled tube. Figure 8 displays the results of the mesh test. A speed of 10 m/s was used for the parametric analysis. Typically, eigenvalue buckling simulation is used to introduce geometrical imperfections into the geometry to account for the effect of geometrical imperfections on the collision behavior of the thin-walled tube. A trial and error method is then used to size the first order buckling modes to 2.00% of the tube thickness.

**Figure 9:**
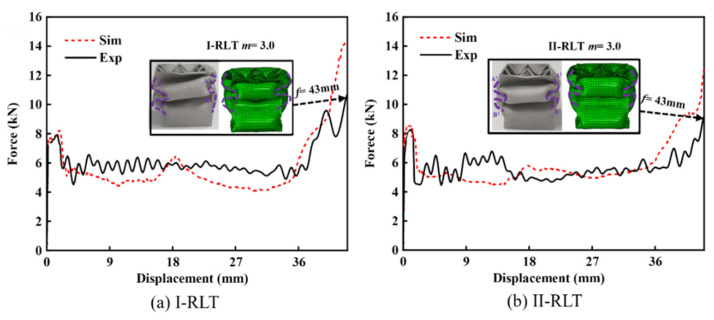
Experimental and finite element results for thin-walled tubes reinforced with gradient lattices.

To assess the validity of the simulation model for the gradient lattice-reinforced thin-walled tube, we established a model with the same structural parameters as the test specimen and conducted finite element simulation. The load–displacement curves of the numerical simulation and test are shown in Figure 9, as well as the deformation of the specimen. The results indicate that the numerical simulation is in good agreement with the test results.

The specimen underwent impact loading and formed two complete folds, corresponding to the two peak load values on the load–displacement curve. The point-reinforced thin-walled tubes in both gradient modes belong to the symmetric deformation mode. Figure 9 shows the deformation diagram of the test with an effective impact distance of 43 mm. It can be observed that after the formation of the second folding of the hybrid tube, a new folding process takes place, resulting in a small deformation and incomplete densification. Simultaneously, outward or inward folds were formed on the two opposite faces (folds A and B), but the folds formed in opposite directions on the two adjacent faces. It has been observed that the I-RLT produces a slight tilt. This is caused by the force exerted by the punch on the upper end of the member, resulting in uneven stress on the filler tube. When the punch impacts at a certain speed, there is a slight difference in the formation time of the folds on opposite faces. This is indicated by the different locations of the start of the folds. Eventually, deformation occurs as shown in Figure 9a.

There is a slight difference between the results obtained from tests and numerical simulations. The comparison of crashworthiness indicators is shown in Table 4. The errors for *EA* and *PCF* are only about 6.0% and 2.0%. The frequency and amplitude of the impact curve in the fluctuation section are high due to the strong and short-lasting impact load. This load is accompanied by the propagation, reflection and projection of compressive stress waves, which cause dynamic buckling of the structure. As a result, the load value fluctuates more than in the simulation. However, during the impact test, the punch rapidly descends from a certain height, and the impact is caused by the self-weight of the platen and resulting impact speed. When the punch makes contact with the member, there may be some instability. The end result is that these small instabilities do not affect the energy absorption of the construction. The numerical simulation places the top rigid plate directly above the structure. The impact effect is achieved by setting a certain compression distance, which completely avoids the instability phenomenon.

## 3. Results and Discussion

### 3.1. Force–Displacement Curves

**Figure 10:**
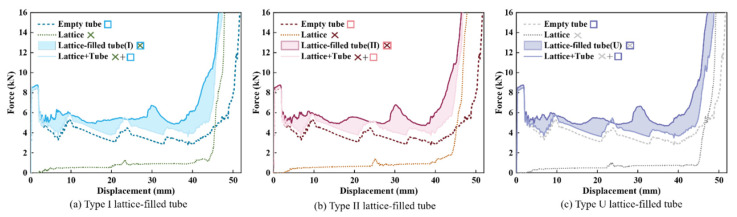
Load–displacement curves of lattice-filled tubes and their components.

**Figure 11:**
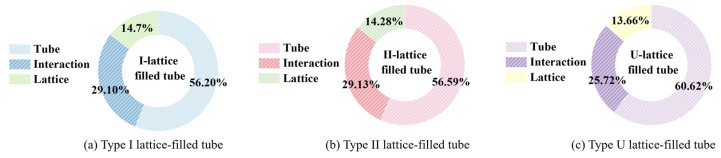
Ratio of energy absorption of different components to total energy absorption.

Examples of force–displacement curves and energy absorbed by each component for type I gradient lattice-filled tubes, type II gradient lattice-filled tubes and uniform lattice-filled tubes under impact loading are shown in Figure 10 and Figure 11. The area of increased energy absorption due to the interaction between the tube wall and the lattice is the area between the ‘lattice + tube’ and the filled tube. As can be seen in Figure 10, filling the thin-walled tube with a gradient lattice significantly improves the crashworthiness of the thin-walled structure. After calculation, it was obtained that the *SEA* of type I gradient lattice-filled tube and type II gradient lattice-filled tube was 24.36% and 22.82% higher than that of empty tube. The overall energy absorption of the filled tube is higher than the sum of the lattice and the empty tube. In order to further indicate the proportion of the total energy absorption of each component to the total energy absorption, Figure 11 has been plotted for observation. It can be seen that the absorbed energy contributed by the gradient lattice is higher than that of the uniform lattice, while the tube wall/lattice interaction is improved by about 4.00% compared to the uniform lattice-filled tube, which shows that the gradient lattice embedded in the thin-walled tubes has a much better crashworthiness and the advantage can be increased in the future by adjusting the gradient coefficients.

### 3.2. Effect of Gradient Patterns on Crashworthiness

**Figure 12:**
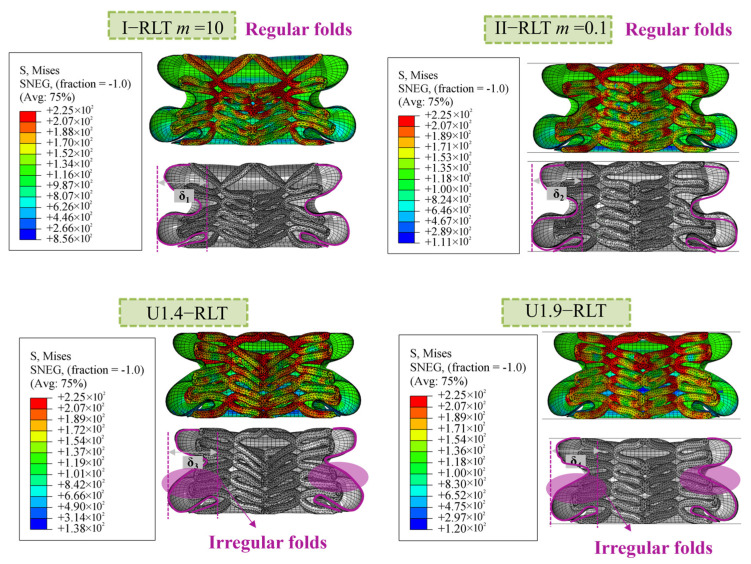
Comparison of deformation of thin-walled tubes reinforced with gradient lattices and thin-walled tubes reinforced with uniform lattice.

The gradient lattice filling has a considerable effect on the folding mode of the hybrid structure. Figure 12 compares the deformation of gradient lattice-reinforced thin-walled tubes and uniform lattice-reinforced tubes with maximum diameter (*D_max_*) and minimum diameter (*D_min_*) of 1.9 mm and 1.4 mm, respectively, a wall thickness of 1.0 mm and an impact velocity of 10 m/s. The observed deformation is the deformation at an effective impact distance of *f* = 48 mm, and the two groups of filled tubes with larger deformation giants are selected for comparison. The figure shows that gradient lattice-reinforced thin-walled tubes (I-RLT and II-RLT) have stable and uniformly long folds, whereas uniform lattice-reinforced thin-walled tubes (U1.4-RLT and U1.9-RLT) have folds of varying lengths and incomplete deformations at the local level. This ultimately results in unsatisfactory energy absorption of the entire structure. Therefore, when the range of peak load is small, it is recommended to select I-RLT with a gradient index *m* less than 1. Conversely, when the range of peak load is large, it is recommended to select I-RLT with a gradient index *m* greater than 1. The type I and type II gradient modes enhance the crashworthiness of the hybrid structure, making them potential energy-absorbing structures when compared to conventional uniform tubes.

**Figure 13:**
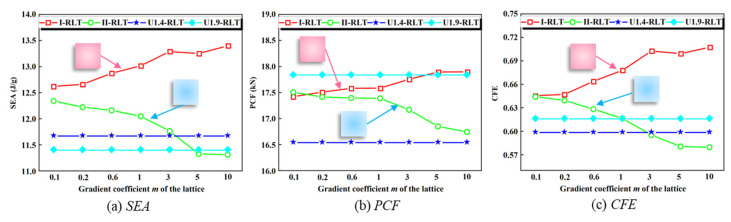
Comparison of *SEA*, *PCF* and *CFE* of thin-walled tubes reinforced with gradient lattices and thin-walled tubes reinforced with uniform lattice.

When the gradient coefficient *m* is 0, the lattice of uniform rod diameters is obtained with diameter values of 1.9 mm and 1.4 mm, respectively. The *SEA*, *PCF* and *CFE* of three types of lattice-reinforced thin-walled tubes are shown in Figure 13: I-RLT, II-RLT, U1.4-RLT and U1.9-RLT. As shown in Figure 13a, the *SEA* of the I-RLT gradually increases with increasing gradient coefficient *m*, and the *SEA* of the II-RLT gradually decreases with increasing gradient coefficient *m*. As shown in Figure 13b, the *PCF* and *SEA* have the same trend, which interferes with the judgement of whether the energy-absorbing structure is excellent in terms of crashworthiness, so it is necessary to prefer the structure through the complex ratio assessment method. It is noteworthy that the I-RLT has a 1.17 times higher *SEA* than the U1.9-RLT, while a lower peak load is obtained. It is evident that the I-gradient mode has a better enhancement effect. As shown in Figure 13c, the *CFE* of the I-RLT shows a gradual increase and produces a larger *CFE* than that of the II-RLT and the U-RLT. The *CFE* of the II-RLT shows an opposite trend to that of the I-RLT, and the *CFE* of the II-RLT is gradually lower than that of the U-RLT when the gradient index *m* is greater than 3.

### 3.3. Effect of Mass Ratio on Crashworthiness

**Figure 14:**
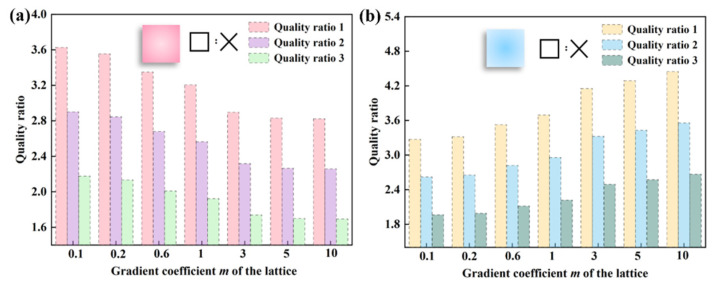
Mass ratio of gradient lattice-filled tubes. (**a**) I-RLT (**b**) II-RLT.

**Figure 15:**
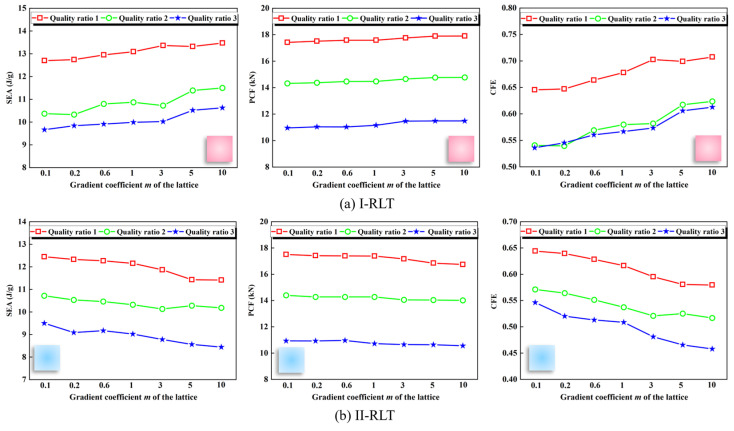
*SEA*, *PCF* and *CFE* of thin-walled tubes reinforced with gradient lattices at different quality ratios.

This section investigates the effect of the ratio of thin-walled mass to lattice mass on the crashworthiness of hybrid structures. The study focuses on *D_max_* and *D_min_* of 1.9 mm and 1.4 mm, respectively, and an impact velocity of 10 m/s. The mass ratios for categories 1, 2 and 3 at different gradient indices are as follows: the data for mass ratio 1 are 1.25 times higher than for mass ratio 2, and 1.67 times higher than for mass ratio 3. The values are shown in Figure 14. The mass ratio of I-RLT decreases gradually while that of II-RLT increases gradually. The trends of *SEA*, *PCF* and *CFE* with increasing gradient index for each mass ratio of I-RLT and II-RLT are shown in Figure 15.

The crashworthiness parameters of the I-RLT gradually decrease with decreasing mass ratio at the same gradient index, as shown in Figure 15a. At different gradient indices, the crashworthiness of I-RLT gradually increased with decreasing mass ratio. The parameters of II-RLT gradually decreased with decreasing mass ratio at the same gradient index from Figure 15b. The *SEA*, *PCF* and *CFE* of II-RLT decreased gradually with decreasing mass ratio at different gradient indices. It was found that the *PCF* shows the same trend when the *SEA* varies along with the variation of *SEA*, irrespective of the gradient pattern. The optimum mass ratios for both I-RLT and II-RLT are located in the case where the gradient index and the thin-wall mass are large.

### 3.4. Effect of Diameters Range on Crashworthiness

**Figure 16:**
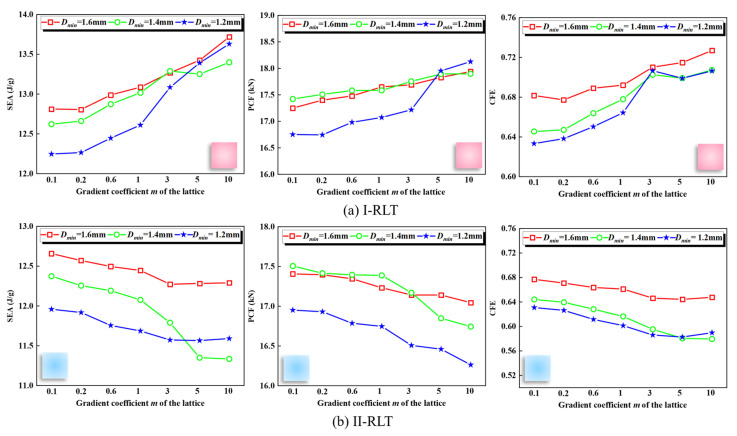
*SEA*, *PCF* and *CFE* of thin-walled tubes reinforced with gradient lattices at different *D_min_*.

To investigate the impact resistance of gradient lattice-reinforced thin-walled tubes, the effect of diameters range was examined. The values of *D_max_* and wall thickness were 1.9 mm and 1.0 mm, respectively, while the impact velocity was 10 m/s. Three different values of *D_min_* (1.6 mm, 1.4 mm and 1.2 mm). The *SEA*, *PCF* and *CFE* of I-RLT at different gradient indices *m* are shown in Figure 16a. The results indicate that the diameters range has a significant impact on the structural crashworthiness. Increasing *D_min_* leads to larger *SEA* and *CFE* values, while the impact of diameters range on *PCF* is equally significant. The *PCF* of the I-RLT with *D_min_* of 1.2 mm is higher than the other two ranges when *m* is greater than 5. On one hand, the total mass of the filled tube differs by no more than 15% for structures with a larger gradient index, regardless of the minimum diameters of the lattices. This results in a negligible difference in the values of *SEA*, *PCF* and *CFE*. The *SEA*, *PCF* and *CFE* of II-RLT at different gradient indices *m* are shown in Figure 16b. The *SEA* and *CFE* increase as *D_min_* increases. For *m* greater than 3, the *SEA* and *CFE* of the II-RLT structure with a minimum diameter of 1.2 mm exhibit an upward trend, while the *PCF* remains at a lower level.

### 3.5. Effect of Impact Velocity on Crashworthiness

**Figure 17:**
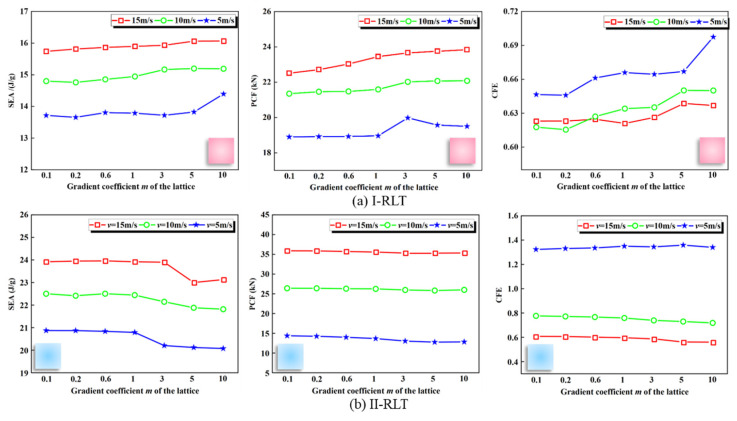
*SEA*, *PCF* and *CFE* of thin-walled tubes reinforced with gradient lattices at different velocities.

To investigate the impact resistance of gradient lattice-reinforced thin-walled tubes at low impact velocity, we simulated the strain-rate effects of Al-1060 and AlSi10Mg using the Cowper–Symonds power law of superstress and the Johnson–Cook plasticity model, respectively. The material constants *D* and *n* in the Cowper–Symonds power law of superstress were taken as 128,800 s^−1^ and 4 [30,31], and the material constants *C* and in the Johnson–Cook plasticity model are taken as 0.02 and 0.001 s^−1^, respectively [22,32]. The impact resistance of the finite element numerical model was analyzed for three different impact velocities: *v* = 5 m/s, *v* = 10 m/s and *v* = 15 m/s. The tubes have diameter values of 1.9 mm and 1.4 mm, respectively, and a wall thickness of 1.0 mm, and a trend plot of the impact resistance indexes was obtained as shown in Figure 17.

The *PCF* and *CFE* of the I-RLT are consistently smooth across different velocities as shown in Figure 17a. When *m* was greater than 3, the *PCF* remained unchanged. This is because the *PCF* is closely related to the wall thickness and can be greatly affected by changes in wall thickness. Currently, all wall thicknesses are 1.5 mm, indicating an insignificant change in *PCF*. The *PCF* and *CFE* of the II-RLT are relatively smooth, with the highest load impact efficiency at a speed of 5 m/s, as shown in Figure 17b. In addition, the *PCF* and *CFE* of the II-RLT are smoother than those of the II-RLT. When the impact velocity was tripled, the *SEA* and *PCF* increased by 14.60% and 22.98%, and 14.80% and 19.10%, respectively. For aluminum alloy materials that are insensitive to strain rate, the change in energy absorption during low-velocity impact loading is not significant, which is consistent with the findings of Langseth et al. [33]. However, the hybrid structure is subject to inertial effects, which affect the crashworthiness index.

### 3.6. A Parametric Study Based on the Complex Proportional Assessment (COPRAS)

The objective of crashworthiness design is to achieve structures with high *SEA*, high *CFE* and low *PCF*. However, it is often observed that as the absorbed energy increases, the maximum peak tends to be higher, indicating that achieving high *SEA* and low *PCF* conditions can be conflicting goals. Multi-attribute decision making (MADM) techniques have been proposed by experts and academics to aid decision making. These techniques include the Technique of Sequential Preference Similarity for Ideal Solutions (TOPSIS), the Preference Ranking Organizational Approach for Enrichment Evaluation (PROMETHEE), the Hierarchical Analysis Method (AHP) and Complexity Proportionality Assessment Method (COPRAS) [34]. Because of its simplicity and accuracy, the COPRAS method is widely used for the identification of thin-walled tubes with an optimal response to energy absorption. The design procedure gives priority to the design objectives on the basis of their ability to absorb energy in the following sequence.

Step 1: Draw up an initial matrix of the options, *A*.

Attributes that reflect the energy-absorption response are shown in the matrix *A*:

(8) $$A={\left[a_{ij}\right]}_{\alpha \beta }=\left[\begin{array}{cccc} a_{11} & a_{12} & \cdots & a_{1\beta }\\ a_{21} & a_{22} & \cdots & a_{2\beta }\\ \cdots & \cdots & \cdots & \cdots \\ a_{\alpha 1} & a_{\alpha 2} & \cdots & a_{\alpha \beta } \end{array}\right]$$

The equation above shows the performance of the first tube in relation to the first attribute, where α is the number of objects observed and β is the number of attributes.

Step 2: Create the normalization matrix *C*.

As the various design attributes have varying units, they cannot be directly compared with each other and this makes the selection process much more difficult. Thus, the matrix *A* should be converted to one without dimensions in order to compare the design attributes. The matrix *C* can have the expression:

(9) $$C={\left[c_{ij}\right]}_{\alpha \beta }=\frac{a_{ij}}{\sum_{i=1}^{\alpha }A_{ij}}$$

where $c_{ij}$ denotes the first normalized attribute of the first design object.

Step 3: Calculation of individual weights for each attribute.

To begin, compare every two attributes at a time. This will result in a summed comparison equal to N=(β(β−1)/2). When comparing two attributes that have different levels of importance in the selection process, assign three points to the more important attribute and one point to the less important attribute. Two points are allocated to each of two equally important attributes during the selection process. This ensures an equal distribution of points for each attribute:

(10) $$W_{j}=\sum_{i=1}^{N}w_{ij}$$

The equity weights for each attribute are shown in Table 5. The study evaluated the attributes *EA*, *SEA*, *PCF*, *MCF* and *CFE*. *EA*, *SEA* and *PCF* were deemed more important than *MCF* and *CFE*. Divide the total score of each attribute *j*_th_ by the total global score to give the weight of the attribute:

(11) $$w_{j}=\frac{W_{j}}{G}=\frac{W_{j}}{\sum_{j=1}^{\beta }W_{j}}$$

Step 4: Calculate the weighted normalization matrix *D*.

By multiplying the matrix *R* by the weights of each attribute and then applying a normalized weighted matrix, the matrix *D* is generated.

(12) $$D={\left[y_{ij}\right]}_{\alpha \beta }=r_{ij}\times w_{j}$$

Step 5: Calculate the total contributions of each design object, including both beneficial and non-beneficial aspects.

There are beneficial (+*y_ij_*) and non-beneficial (−*y_ij_*) contributions in matrix *D*. Beneficial Contributions are attributes that benefit the design object and are referred to as *EA*, *SEA*, *MCF* and *CFE*. *A* higher *PCF* is not beneficial to the design object and is not considered to be a helpful input. Use variables *S_+i_* and *S_−i_* to represent the total contributions, including both beneficial and non-beneficial ones:

(13) $$S_{+i}=\sum_{j=1}^{n}+y_{ij}$$

(14) $$S_{-i}=\sum_{j=1}^{n}-y_{ij}$$

Step 6: Determination of the minimum value of the contribution that is not productive, denoted as $S_{-\min}=\min S_{-i}$.

Step 7: Calculate the relative value ($Q_{i}$) and quantitative benefits ($U_{i}$) of each design object. 

The ranking shows the terms for maximum and minimum relative terms first and last:

(15) $$Q_{i}=S_{+i}+\frac{S_{-\min}\times \sum_{i=1}^{m}S_{-i}}{S_{-i}\times \sum_{i=1}^{m}\left(S_{-\min}/S_{-i}\right)}$$

(16) $$U_{i}=\frac{Q_{i}}{Q_{\max}}\times 100$$

**Figure 18:**
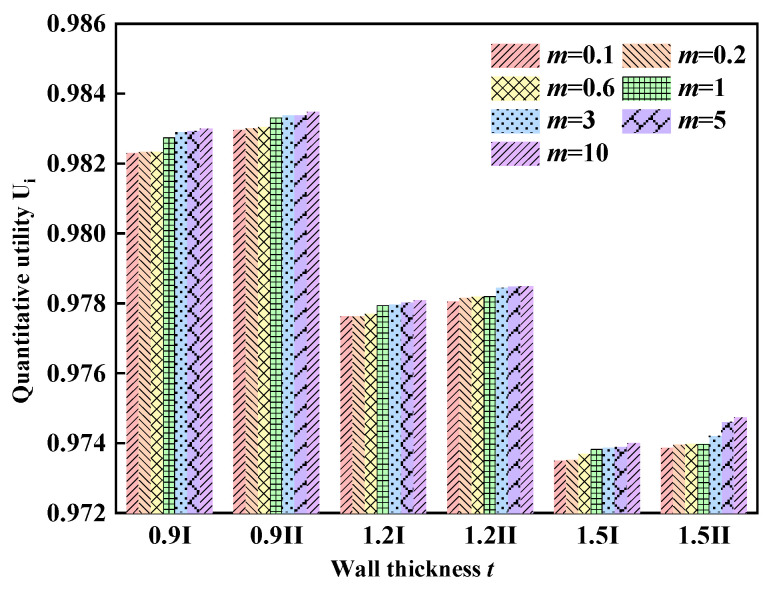
Evaluation results for complex proportions.

Table 6 displays the property parameters and final rankings of Type I gradient and Type II gradient mode filled tubes with varying wall thicknesses. Figure 18 shows the results of the quantitative utility. It is evident that the type II gradient mode filled tube with a wall thickness of 0.9 mm and a gradient coefficient of 10 is the best structure, while the type I gradient mode filled tube with a wall thickness of 1.5 mm and a gradient coefficient of 0.1 is the worst. Additionally, it is observed that the crashworthiness of the filled tubes improves as the gradient index increases. Although increasing the wall thickness significantly enhances the energy absorption of the structure, it does not improve the overall crashworthiness.

## 4. Conclusions

This study proposes two types of gradient lattice-reinforced thin-walled structures. The study confidently investigated the crashworthiness of the hybrid structure through a combination of experimental and numerical simulation methods. The analysis included the effects of lattice gradient pattern, wall thickness, diameters range and impact velocity. The results indicate that:

(1) Introducing the gradient design allows the energy-absorption efficiency of the thin-walled structure to be effectively improved while not significantly increasing the maximum crushing force. The thin-walled tube reinforced with inward radial gradient lattice has a specific absorption energy 17.44% higher than that of the thin-walled tube reinforced with a uniform lattice, while the *PCF* remains almost unchanged.
(2) The gradient pattern with the radius decreasing inwardly is superior to that with the radius decreasing outwardly under impact loading, and is also better than that of the uniform lattice-reinforced thin-walled tube.
(3) The strength and stability of the pattern shifting of a reinforced thin-walled tube with a gradient lattice structure are better than before. It interacts more forcefully with the wall, resulting in enhanced specific absorption energy.
(4) A comprehensive evaluation of the crashworthiness of the structures using the composite proportionality evaluation method showed that the crashworthiness of the gradient lattice-filled tubes improved as the gradient index approached 10. Of the design parameters studied, the structures with *t* = 0.9 mm and *m* = 10 mm had excellent crashworthiness.

## Figures and Tables

**Table 1:** Structure naming.

Items	*m*	*R_min_* (mm)	*R_max_* (mm)	Number of Cells	Design Quality (g)	Reality Quality (g)	Quality Errors (%)
I-RL	3	0.45	0.81	32	8.74	9.46	8.23
II-RL	3	0.7	0.95	32	8.74	9.44	8.01

**Table 2:** AlSi10Mg specimen process parameters.

Laser Power/W	Scan Speed/(m/s)	Hatch Spacing/mm	Layer Thickness/mm	Ambient Temperature (°C)
(500 W/1000 W) × 4	4.00	0.19	0.05	25

**Table 3:** Mechanical properties of Al-1060 and AlSi10Mg.

Materials	Density (g/cm^3^)	Elastic Modulus *E* (GPa)	Poission’s Ratio	Initial Yield Stress (MPa)	Ultimate Stress (MPa)
AlSi10Mg	2.67	62.9	0.3	125	325
Al-1060	2.70	64.8	0.3	45	118

**Table 4:** Crashworthiness metrics for experimental and numerical simulations.

Items	*EA* (J) (Results of Experiment)	*EA* (J) (Results of Simulation)	*PCF* (kN) (Results of Experiment)	*PCF* (kN) (Results of Simulation)	*MCF* (kN) (Results of Experiment)	*MCF* (kN) (Results of Simulation)
I-RLT *m* = 3.0	180.20	166.84	8.05	8.22	4.19	3.88
II-RLT *m* = 3.0	180.80	176.53	8.25	8.29	4.20	4.11

**Table 5:** Weights for each attribute.

	1	2	3	4	5	6	7	8	9	10	*W_j_*	*w_j_*
*EA*	2	2	3	3							10	0.25
*SEA*	2				2	3	3				10	0.25
*PCF*		2			2			3	3		10	0.25
*MCF*			1			1		1		2	5	0.125
*CFE*				1			1		1	2	5	0.125
Total											40	1

**Table 6:** Calculated data for the complex ratio of gradient lattice-filled tubes.

*t*	*m*	*S_+i_*	*S_−i_*	*S_−min_/S_−i_*	*Q*	*Q*_max_	*U*	RANK
0.9I-RLT	0.1	0.0154	0.0048	0.9204	0.2654	0.2702	0.9823	14
	0.2	0.0163	0.0048	0.9191	0.2663	0.2710	0.9823	13
	0.6	0.0164	0.0048	0.9190	0.2664	0.2712	0.9823	12
	1	0.0151	0.0047	0.9460	0.2651	0.2697	0.9827	11
	3	0.0146	0.0046	0.9563	0.2646	0.2692	0.9829	10
	5	0.0149	0.0046	0.9570	0.2649	0.2695	0.9829	9
	10	0.0143	0.0046	0.9634	0.2643	0.2688	0.9830	7
0.9II-RLT	0.1	0.0136	0.0046	0.9631	0.2636	0.2682	0.9830	8
	0.2	0.0137	0.0046	0.9661	0.2637	0.2682	0.9830	6
	0.6	0.0143	0.0046	0.9659	0.2643	0.2689	0.9830	5
	1	0.0133	0.0045	0.9851	0.2633	0.2678	0.9833	4
	3	0.0123	0.0044	0.9926	0.2623	0.2668	0.9834	3
	5	0.0127	0.0044	0.9914	0.2627	0.2672	0.9834	2
	10	0.0121	0.0044	1.0000	0.2621	0.2665	0.9835	1
1.2I-RLT	0.1	0.0191	0.0062	0.7149	0.2691	0.2752	0.9776	28
	0.2	0.0193	0.0062	0.7146	0.2693	0.2754	0.9776	27
	0.6	0.0179	0.0061	0.7204	0.2679	0.2740	0.9777	26
	1	0.0176	0.0060	0.7296	0.2676	0.2736	0.9779	25
	3	0.0178	0.0060	0.7295	0.2678	0.2739	0.9780	24
	5	0.0167	0.0060	0.7347	0.2667	0.2726	0.9780	23
	10	0.0167	0.0060	0.7371	0.2667	0.2726	0.9781	21
1.2II-RLT	0.1	0.0175	0.0060	0.7332	0.2675	0.2735	0.9780	22
	0.2	0.0165	0.0060	0.7397	0.2665	0.2725	0.9782	20
	0.6	0.0169	0.0060	0.7397	0.2669	0.2729	0.9782	19
	1	0.0172	0.0060	0.7395	0.2672	0.2732	0.9782	18
	3	0.0160	0.0059	0.7512	0.2660	0.2719	0.9784	17
	5	0.0161	0.0059	0.7519	0.2661	0.2720	0.9785	16
	10	0.0159	0.0058	0.7533	0.2659	0.2718	0.9785	15
1.5I-RLT	0.1	0.0242	0.0075	0.5899	0.2742	0.2816	0.9735	42
	0.2	0.0244	0.0075	0.5898	0.2744	0.2819	0.9735	41
	0.6	0.0242	0.0074	0.5945	0.2742	0.2816	0.9737	40
	1	0.0229	0.0073	0.6004	0.2729	0.2802	0.9738	39
	3	0.0233	0.0073	0.6003	0.2733	0.2806	0.9739	37
	5	0.0223	0.0073	0.6029	0.2723	0.2796	0.9739	36
	10	0.0222	0.0073	0.6059	0.2722	0.2795	0.9740	32
1.5II-RLT	0.1	0.0221	0.0073	0.6030	0.2721	0.2794	0.9739	38
	0.2	0.0212	0.0073	0.6071	0.2712	0.2785	0.9739	35
	0.6	0.0215	0.0073	0.6068	0.2715	0.2788	0.9740	34
	1	0.0219	0.0073	0.6061	0.2719	0.2791	0.9740	33
	3	0.0204	0.0072	0.6148	0.2704	0.2776	0.9742	31
	5	0.0196	0.0070	0.6265	0.2696	0.2767	0.9746	30
	10	0.0195	0.0070	0.6304	0.2695	0.2765	0.9747	29

## Data Availability

Data are contained within the article.
